# Preparation, characterization and therapeutic properties of gum arabic-stabilized gallic acid nanoparticles

**DOI:** 10.1038/s41598-020-71175-8

**Published:** 2020-10-20

**Authors:** Abdelkader Hassani, Mohammad Mahdi Sabaghpour Azarian, Wisam Nabeel Ibrahim, Siti Aslina Hussain

**Affiliations:** 1 Department of Chemical and Environmental Engineering, Faculty of Engineering, Universiti Putra Malaysia, Serdang, UPM, Serdang, 43400 Malaysia; 2 UPM-MAKNA Cancer Research Laboratory, Institute of Bioscience, Universiti Putra Malaysia, Serdang, UPM, Serdang, 43400 Malaysia; 3 Department of Biomedical Sciences, College of Health Sciences, QU Health, Qatar University, Doha, Qatar

**Keywords:** Cancer, Drug discovery

## Abstract

Gallic acid (GA) is a natural phenolic compound with therapeutic effects that are often challenged by its rapid metabolism and clearance. Therefore,  GA was encapsulated using gum arabic into nanoparticles to increase its bioavailability. The formulated nanoparticles (GANPs) were characterized for physicochemical properties and size and were then evaluated for antioxidant and antihypertensive effects using various established in vitro assays, including 1,1-diphenyl-2-picrylhydrazyl (DPPH), nitric oxide scavenging (NO), β-carotene bleaching and angiotensin-converting enzyme (ACE) inhibitory assays. The GANPs were further evaluated for the in vitro cytotoxicity, cell uptake and cell migration in four types of human cancer cell lines including (MCF-7, MDA-MB231) breast adenocarcinoma, HepG2 hepatocellular cancer, HT-29 colorectal adenocarcinoma, and MCF-10A breast epithelial cell lines. The GANPs demonstrated potent antioxidant effects and have shown promising anti-cancer properties in a dose-dependent manner with a predilection toward HepG2 and MCF7 cancer cells. The uptake of GANPs was successful in the majority of cancer cells with a propensity to accumulate in the nuclear region of the cells. The HepG2 and MCF7 cancer cells also had a significantly higher percentage of apoptosis and were more sensitive to gallic acid nanoparticle treatment in the cell migration assay. This study is the first to confirm the synergistic effects of gum arabic in the encapsulation of gallic acid by increasing the selectivity towards cancer cells and enhancing  the antioxidant properties. The formulated nanoparticles also had remarkably low toxicity in normal cells. Based on these findings, GANPs may have promising therapeutic applications towards the development of more effective treatments with a probable targeting precision in cancer cells.

## Introduction

Cancer is implicated in the top lists of disease-specific mortality rates in developed and developing countries. This disorder results from a genetic mutation in cells causing uncontrolled growth of cell clones capable of circumventing all the preventive regulatory mechanisms due to the privileges provided by the mutation^1^. Although different mechanisms are involved in the etiology of cancer; however, most of the impact is attributed to the presence of reactive oxygen species inside the cells leading to the DNA mutation with its deleterious consequences^2^. Oxidative stress is the phenomenon resulting from the physiological imbalance leading to the accumulation of free radical series of oxygen inside the cells including hydrogen peroxide and superoxide radicals^3^. Moreover, the interactions between molecular oxygen and biomolecules in the biological systems produce harmful free radicals that can cause metabolic perturbations^4^. The active oxygen produced can also interact in response to adequate transition metal catalyst and produce oxidizing species and hydroxyl radicals with high toxicity^5^. Accordingly, many traditional medicines of plant origin were found to be therapeutic in cancer disease due to the antioxidant properties of its constituents, their relative safety, and low toxicity compared with chemotherapeutic agents. This led to the determined quest for drug discovery from different medicinal plant extracts.

Gallic acid (GA) is a natural phenolic compound commonly found in gallnuts, tea leaves, and some fruits with pharmaceutical applications in cancer, microbial infections, and cardiovascular diseases^6^. It is also hepatoprotective and antihyperglycaemic^7^. like many natural antioxidants, it is a potent scavenger of trichloromethyl radicals that prevents lipid peroxidation through the generation of tocopheryl radicals scavengers; it also averts H_2_O_2_ induced lipid peroxidation as well as the production of OH-deoxyguanosine^8^. Despite having all the aforementioned therapeutic properties, gallic acid use is restricted by its poor absorption, rapid elimination, and low bioavailability^9,10^. These obstacles could be resolved with the help of nanoformulation. Nanomedicine field has grown considerably in the last decade with interests of overcoming challenges in drug delivery to the target tissue, increasing the bioavailability and enhancing the targeted uptake of active therapeutic compounds^11,12^. Indeed, the advances in drug design and nanomaterial sciences have developed novel nanoscale drug delivery systems for targeting approaches that could bring cancer therapy to better heights^13^. Nanoparticles have received considerable attention for the improvement of controlled-release or sustained-release in drug delivery systems due to their low toxicity, biocompatibility, and protection of encapsulated agents^14^. The nanoparticle formulation may even enhance the drug delivery in cancer tissue due to a native property of enhanced permeability in the blood capillaries a property known as the enhanced permeability and retention (EPR) effect^15^. These effects may potentiate the antioxidant activity of medicinal active compounds especially if combined with other potent antioxidants used as a coating material in the nanoformulation such as Gum arabic.

Gum arabic is a natural extract from *Acacia* species that include a blend of polysaccharide and glycoprotein phytochemicals^16^. It is used as an emulsifier or stabilizer in various biomedical applications. This plant-based hydrocolloid is traditionally known for its therapeutic properties in diseases such as diabetes mellitus, stroke, and hypertension^16^. Among its many properties, its strong antioxidant property is the most documented effect^17^.

Therefore, a novel nanoparticle system was developed using gum arabic as a coating material to improve the therapeutic efficacy of gallic acid against cancer cells. As far as the objective is concerned, the current study is the first of its kind in the current literature concerned with the antioxidant potential and antihypertensive activity of gallic acid nanoparticles (GANPs). In this study, the pure GANPs have been prepared using the freeze-drying technique. The antioxidant and the antihypertensive activities of gallic acid and GANPs were assessed in vitro by free radical scavenging and angiotensin-converting enzyme (ACE) inhibition assays. The cytotoxicity of both free gallic acid (GA) and GANPs at various concentrations was individually determined based on the MTT assay, confocal microscope, and cell migration assay.

## Results

The GANPs have been synthesized successfully based on the freeze-drying method technique. This method resulted in the preparation of a clear suspension of nanoparticles.

### Characterization of GANPs

**Figure 1:**
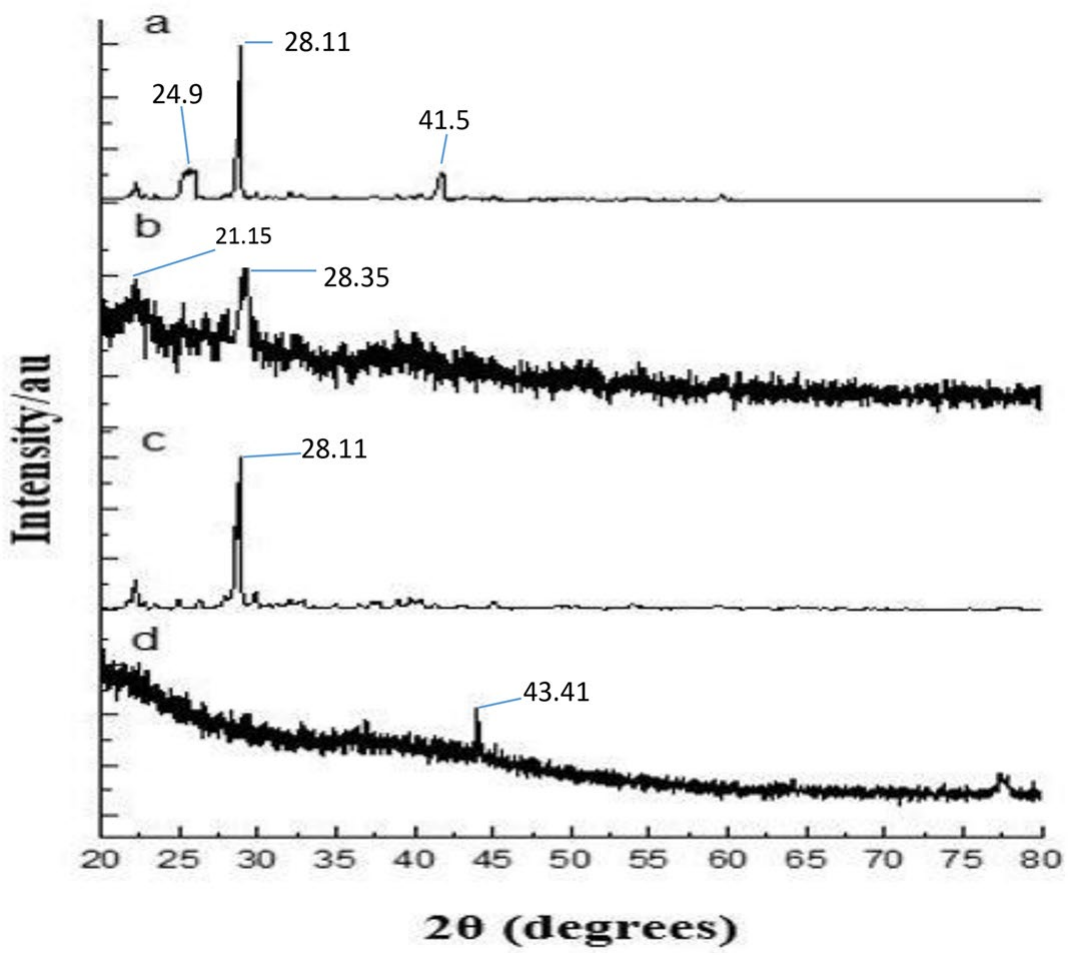
Powder X-ray diffraction patterns of XRD analysis of (**A**) the none capsulated gallic acid, (**B**) Gum arabic, (**C**) Physical mixture of GANPs, (**D**) the nanoparticles of gallic acid prepared using the freeze-drying technique.

**Figure 2:**
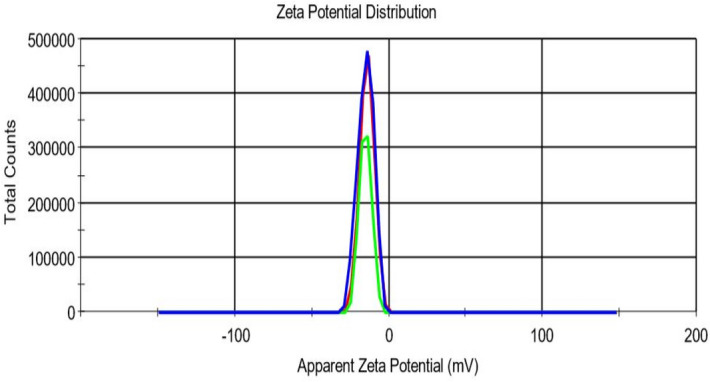
The zeta potential profile of GANPs (− 15.2 mV) produced via freeze-drying method using a zeta sizer Malvern Nano-ZS-ZS, Zeeman.

**Figure 3:**
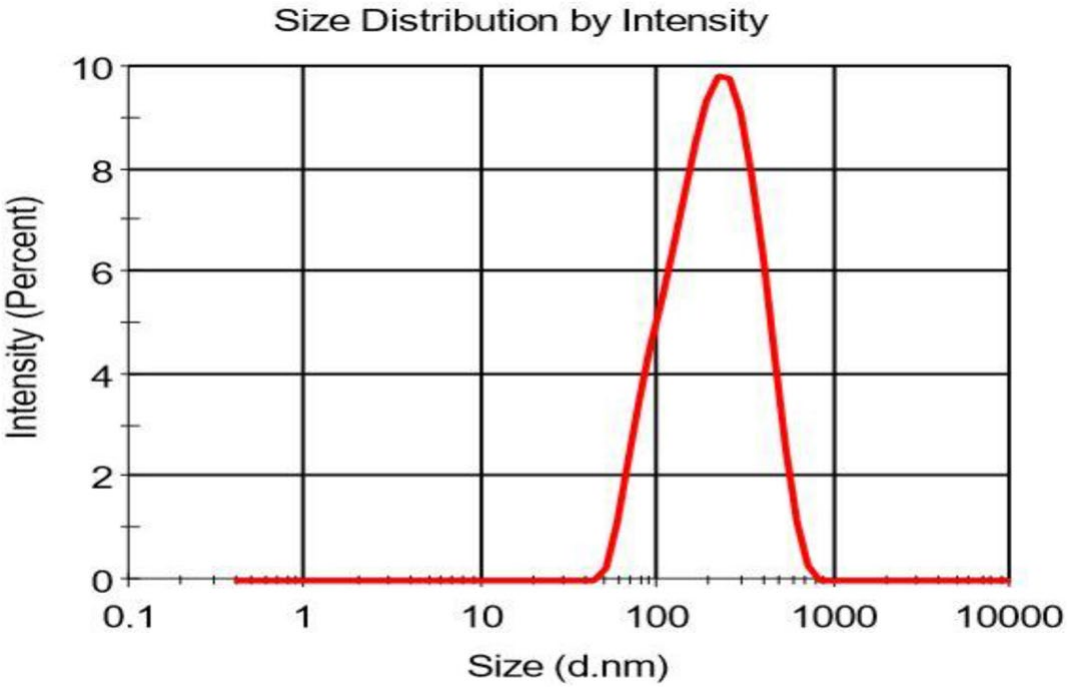
The size distribution of gallic acid encapsulated into gum arabic-nanoparticles in nm size using a zeta sizer Malvern Nano-ZS-ZS, Zeeman with dynamic size.

The X-ray diffraction was performed for (A) GA, (B) gum arabic, (C) physical mixture, and (D) GANPs as illustrated in Fig. 1. In the X-ray diffractogram of gallic acid (Fig. 1a), sharp diffraction peaks were revealed at several diffraction angles including 20.12°, 24.9°, 28.11°, and 41.5°.This diffractogram showed the crystalline nature of gallic acid (GA). The same diffraction peaks revealed in the physical mixture pattern by reduction of intensity (Fig. 1c). It can be shown that the gum arabic peaks depicted at 21.15° and 28.35° are obvious as shown in Fig. 1b. This diffraction indicated that gum arabic was presented as an amorphous material. The particle size of GANPs nanoparticles ranged from 33 to 250 nm, while the zeta potential was − 15.2 mV (Figs. 2, 3).

**Figure 4:**
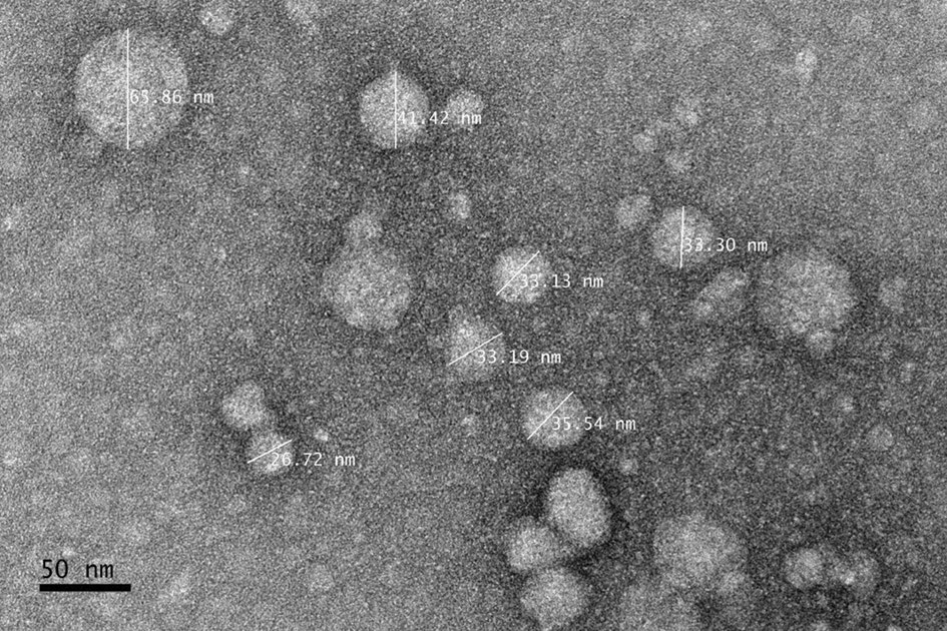
Transmission electron microscopy of GA nanoparticles coated with gum arabic polymer. Demonstrating the spheroid shape with the approximate size of the nanoparticles in nm unit measurement.

The morphology and dispersion of nanoparticles were evaluated using Transmission Electron Microscopy (TEM). TEM image analysis of the developed nanoparticles is depicted in Fig. 4.

**Figure 5:**
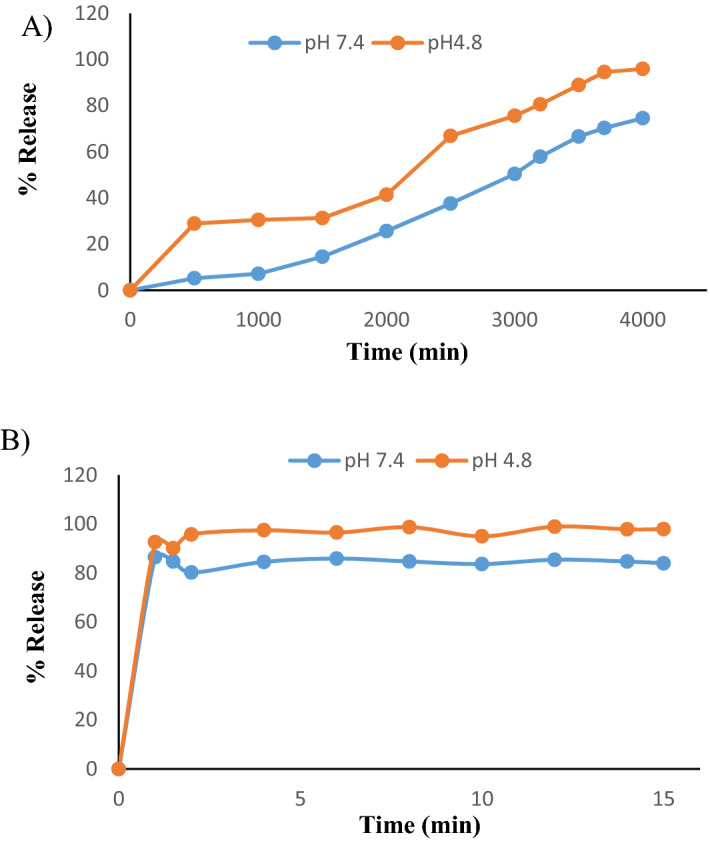
(**A**) Release profiles of GA from GANPs at pH 4.8 and pH 7.4, (**B**) release profiles of GA from its physical mixture at pH 4.8 and pH 7.4.

The release profile of GA from GANPs according to the pH level is demonstrated  in Fig. 5. The amount of GA released from GANPs at the pH level of 4.8 was higher than the amount released in the alkaline pH level of 7.4 and as shown in the figure, the release was approaching completion after 4,000 min.

### Evaluation of the antioxidant properties

**Figure 6:**
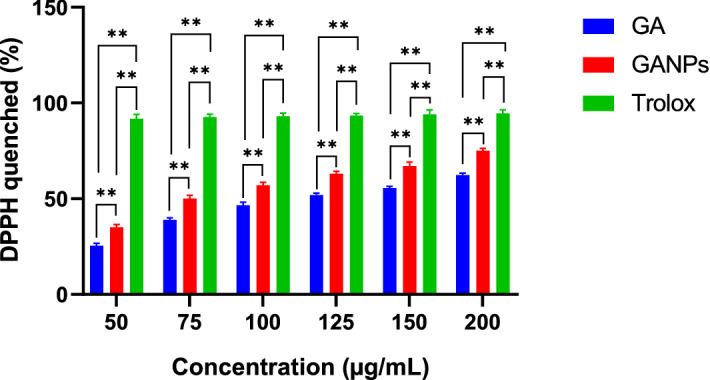
DPPH scavenging activity of free GA and GANPs samples at concentrations ranged from 0–200 µg/mL with statistical analysis using a one-way ANOVA test. Data shown are mean value ± SD (n = 3, *p-value ≤ 0.05, **p-value ≤ 0.01).

The free radical scavenging activity of the free and nano encapsulated gallic acid was assessed in vitro by the DPPH assay. DPPH is a stable organic free radical used to estimate the antioxidant activity of various compounds. Trolox served as a positive control due to its capacity to dissolve in the aqueous system. In this assay, the color of the DPPH changed from deep violet to a pale or colorless solution in the presence of GANPs nanoparticles. As illustrated in Fig. 6, the percentage of DPPH scavenging activity of GANPs was 35.6%, 57.9%, and 75.6% for the concentrations of 50, 100, and 200 µg/mL respectively; whereas for free gallic acid the scavinging activity was 25.4%, 46.6%, and 62.3%, respectively for the same concentrations (*p* < 0.05). This confirms the retention of the complete function of gallic acid after nanoencapsulation in gum arabic nanoparticles*.* Trolox revealed strong scavenging activity of 94.6% against DPPH at similar concentrations.

**Figure 7:**
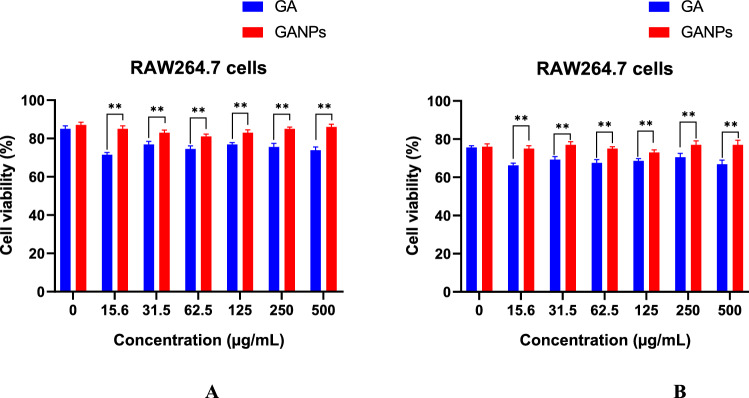
(**A**) Cell viability of RAW 264.7 cells treated with free GA and GANPs for 24 h at various concentrations. (**B**) Cell viability of RAW 264.7 cells treated with free GA, GANPs, and LPS for 24 h. Data shown are mean value ± SD (n = 3, *t* test, *p-value ≤ 0.05, **p-value ≤ 0.01).

**Figure 8:**
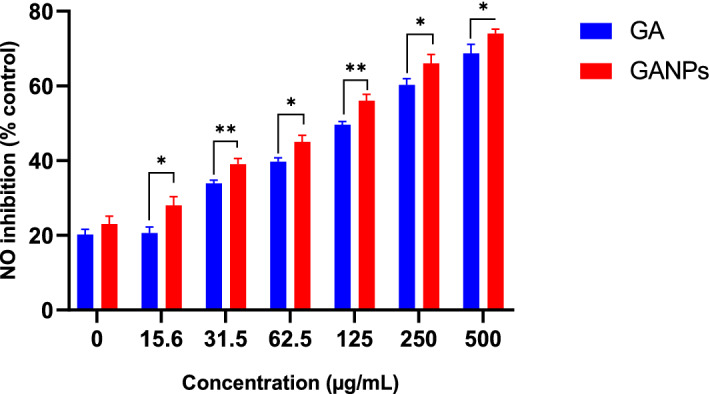
Nitric oxide radical scavenging activities of RAW 264.7 cells treated with different concentrations of free GA and GANPs nanoparticles. Data shown are mean value ± SD (n = 3, *t* test, *p-value ≤ 0.05, **p-value ≤ 0.01).

The antioxidant evaluation of free and nano encapsulated gallic acid was also determined in murine macrophage cell line (RAW 264.7). Initially, the cell viability of RAW 264.7 cells in response to treatments was evaluated using the MTT reduction assay in the presence or absence of LPS. As shown in Fig. 7, the LPS induction had shown a relative toxic effect on the RAW 264.7 cells upon treatment with GA and GANPs. Both treatment forms had negligible cytotoxicity activity against RAW 264.7 cells in comparison to the untreated LPS-stimulated cells. Treatment of RAW 264.7 cells with GA and GANPs caused a considerable inhibition of nitric oxide (NO) production as depicted in Fig. 8. Nonetheless, the nano encapsulated GA exhibited potent antioxidant activity, when compared to the free GA.

**Figure 9:**
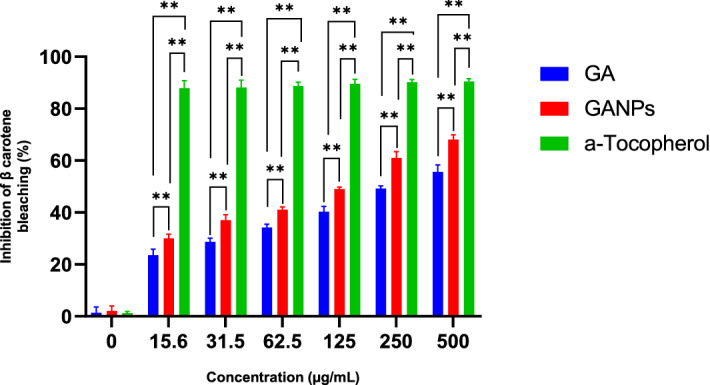
Inhibition (%) of free GA and Gallic acid nanoparticles by β-carotene bleaching assay with statistical analysis using a one-way ANOVA test. Data shown are mean value ± SD (n = 3, *p-value ≤ 0.05, **p-value ≤ 0.01).

The mechanism of the β-carotene bleaching essay is mainly based on the loss of yellow color of β-carotene which could react directly with free radicals produced by the oxidation of linoleic acid in the emulsion. The presence of antioxidants in the reaction mixture is expected retard the reaction (bleaching) of β-carotene. The results demonstrated dose-dependent antioxidant property with GANPs having more potent antioxidant activity than the free GA, as illustrated in Fig. 9.

### The antihypertensive activity of GANPs

**Figure 10:**
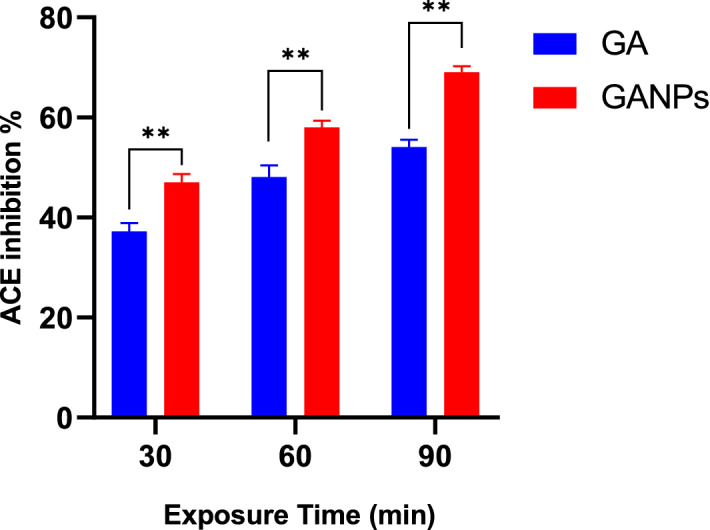
ACE inhibition (%) for free gallic acid and GANPs after 30, 60, and 90 min. Data shown are mean value ± SD (n = 3, *t* test, *p-value ≤ 0.05, **p-value ≤ 0.01).

The in vitro antihypertensive activity of free gallic acid and GANPs was evaluated by measuring the ACE inhibitory activity of the enzyme following the method outlined by Cushman and Cheung^18^. This assay is based on the conversion of hippuryl-histidyl-leucine to hippuric acid. The in vitro antihypertensive capacity of free GA and GANPs was investigated by measuring its absorbance at 228 nm as shown in Fig. 10. The results confirmed the higher ACE inhibitory activity of GANPs compared to free gallic acid.

### Antineoplastic activity of GANPs

**Figure 11:**
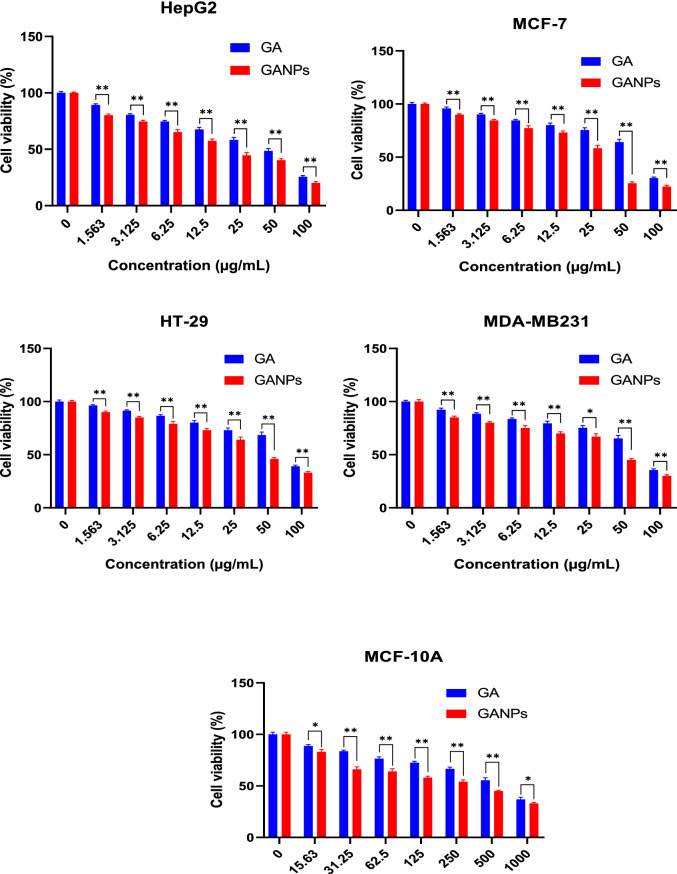
Cytotoxicity activity of GA, GANPs at varying concentrations of 0–100 µg/mL on cancer cell lines (HepG2, HT-29, MCF-7, and MDA-MB231), 0–1,000 µg/mL on normal cell lines (MCF-10A). The cells were incubated for 72 h and the cell viability was assessed by MTT assay. GANPs exhibited selective toxicity towards HepG2 cells. Data shown are mean value ± SD (n = 3, *t* test, *p-value ≤ 0.05, **p-value ≤ 0.01).

**Figure 12:**
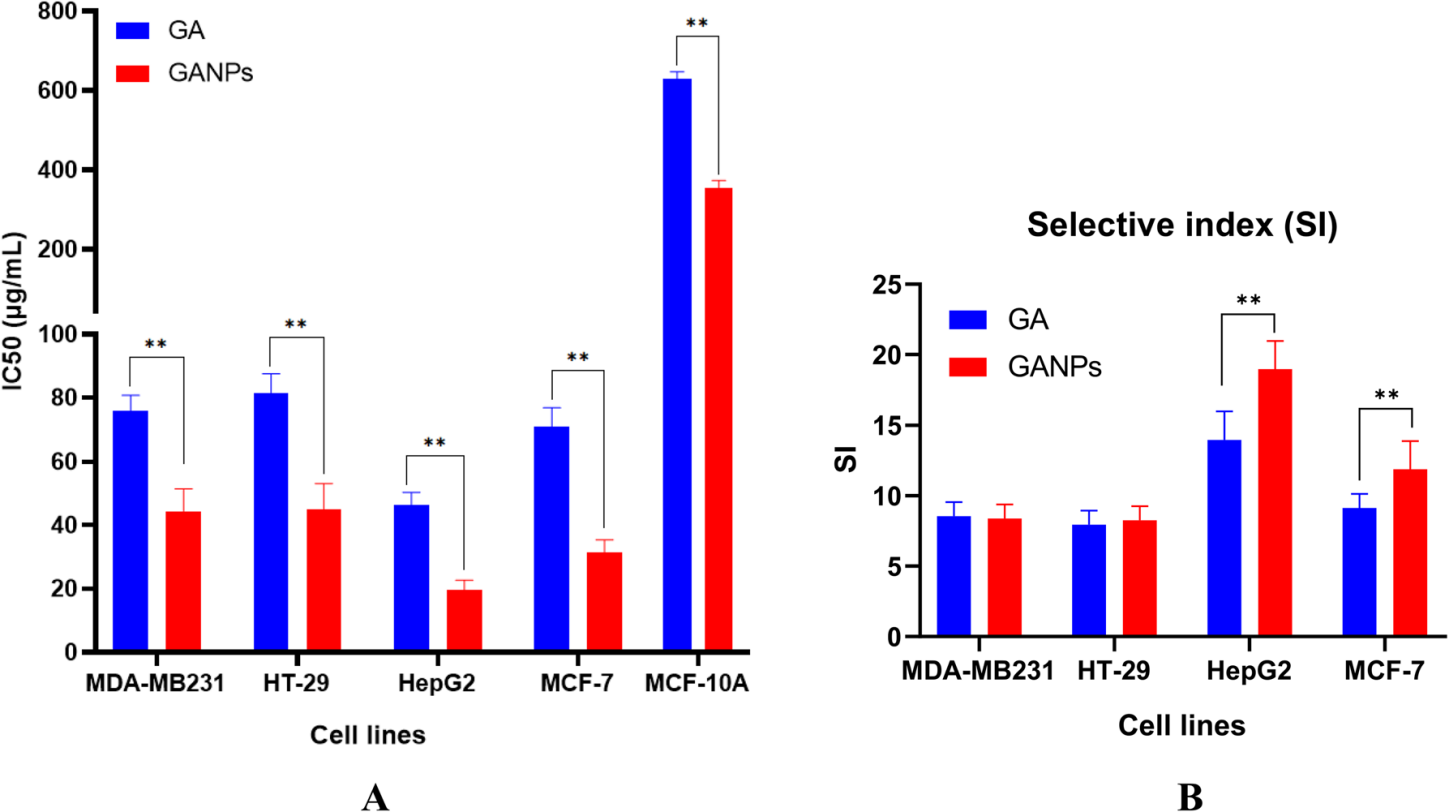
(**A**) illustration of the IC_50_ values of GA and GANPs in different cancer cell lines and normal cells. (**B**) estimation of the selective index based on the IC_50_ values of GA and GANPs with statistical analysis using a one-way ANOVA test. Data shown are mean value ± SD (n = 3, *p-value ≤ 0.05, **p-value ≤ 0.01).

**Figure 13:**
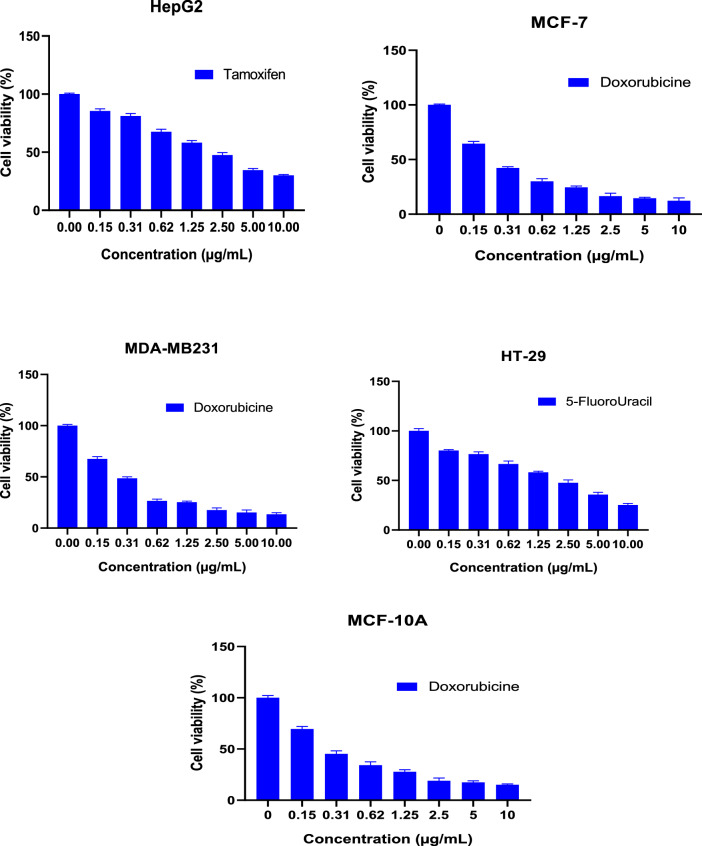
Chemotherapeutic effects on the viability of treated cell lines (HepG2, HT-29, MCF-7, MDA-MB231, and MCF-10A) using MTT assay at varying concentrations of 0–10 µg/mL.

The cytotoxicity of GA and GANPs was assessed in HepG2, MCF7, MDA-MB231, and HT29 cancer cell lines using MTT reduction assay. The cancer cells were treated with free GA and GANPs at concentrations ranging between 1.56 and 100 µg/mL for 72 h. The MCF-10A normal cell line was also included in the treatment with GA and GANPs but with a considerably higher range of concentrations (15.63–1,000 µg/mL) for 72 h. The higher concentration gradient of free GA and GANPs in MCF-10A was used to estimate the IC_50_ and the selective index values as shown in Figs. 11 and 12. The results of the standard chemotherapeutic controls are demonstrated in Fig. 13.

According to the results, significant cytotoxicity was elicited among the HepG2, MCF7, MDA-MB231, and HT29 cell lines after treatment with IC_50 _ concentrations of  free and nano encapsulated gallic acid as shown in Fig. 12. There was negligible cytotoxicity among the MCF-10A cells especially at concentrations equivalent to the IC_50_ concentrations used in cancer cells (Fig. 11) and also in comparison with the untreated MCF-10A cells.

Interestingly, the HepG2 and MCF7 cells were more sensitive to the treatments than the HT29 and MDA-MB231 cells with a significantly higher selectivity with IC_50_ of 16.61 and 8.52 µg/mL for free GA and GANPs respectively as shown in Fig. 12. The IC_50_ value of GANPs was approximately half that of free gallic acid in HepG2 cells as shown in Fig. 12. As demonstrated, the HT29 and MDA-MB231 cells were less sensitive to the treatments with an IC_50_ concentration of 35.45 and 19.62 µg/mL respectively.

**Figure 14:**
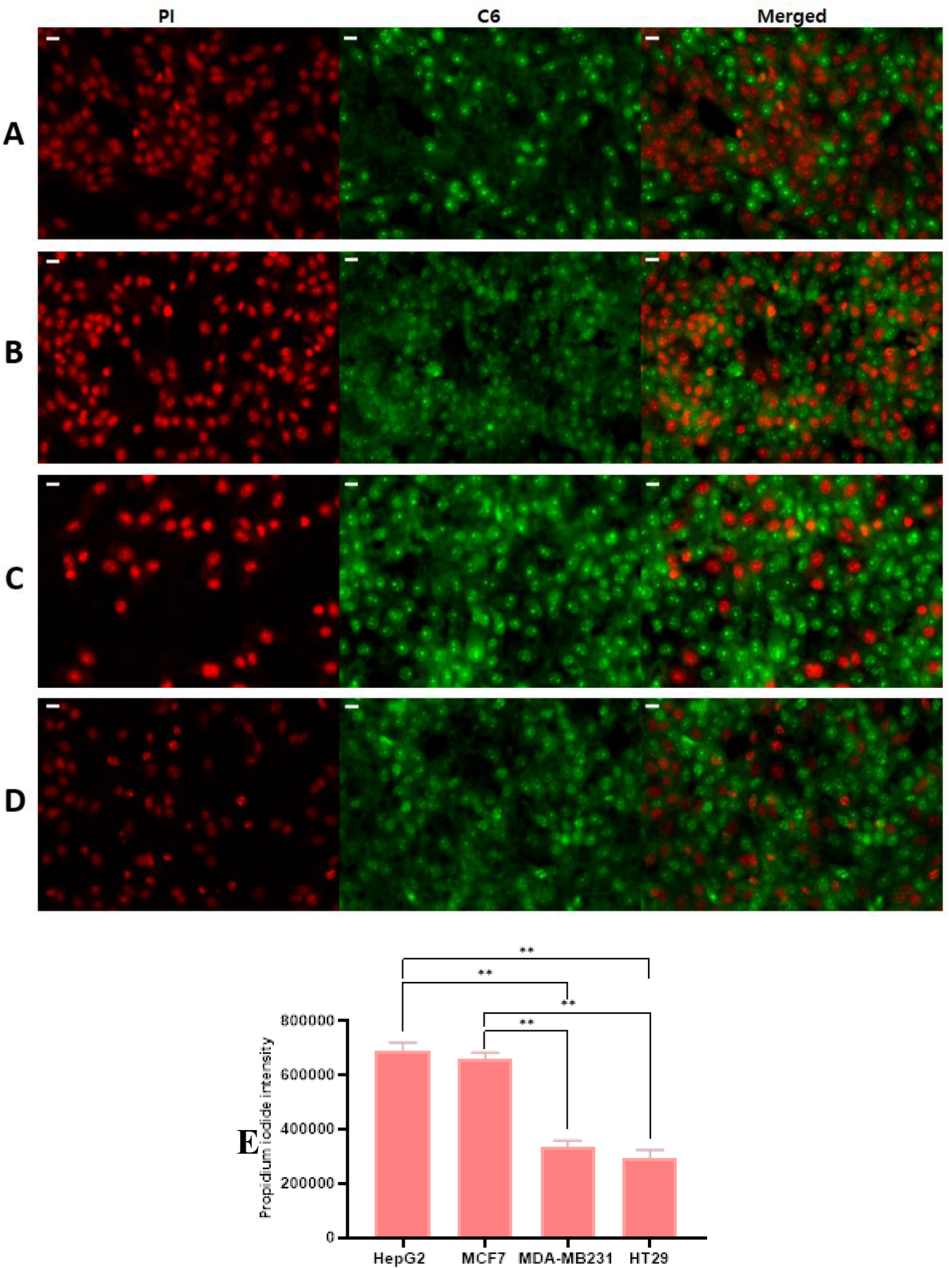
Cell uptake and cytotoxicity studies of GA/C6NPs with IC_50_ values of concentrations using coumarin-6 (C6) as nanoparticle tracker and propidium iodide (PI) as a counterstain for cell viability in (**A**) HepG2, (**B**) MCF7, (**C**) MDA-MB231, and (**D**) HT29 cells for 24 h. The scale bar is 20 µm (**E**) PI fluorescence intensity analysis among the cancer cell lines using ImageJ software; with statistical analysis using a one-way ANOVA test. Data shown are mean value ± SD (n = 3, *p-value ≤ 0.05, **p-value ≤ 0.01).

**Figure 15:**
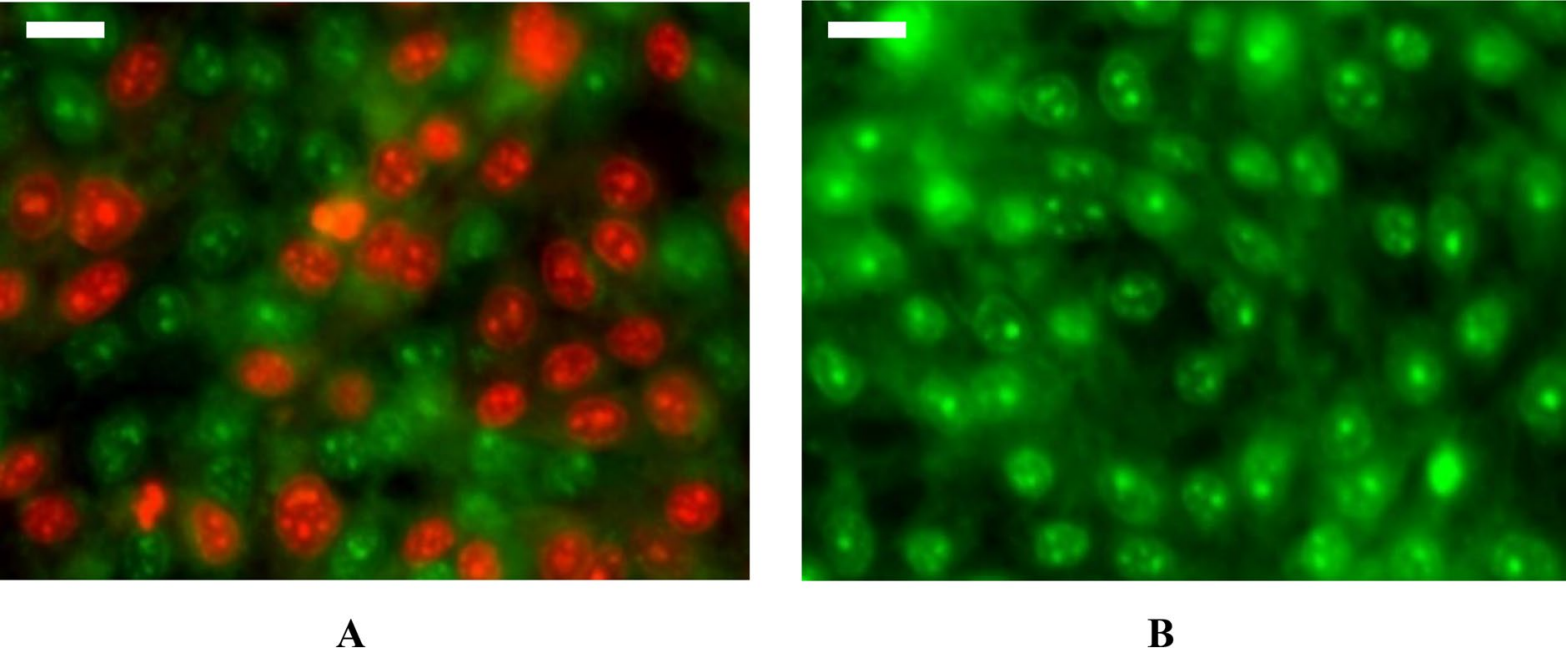
Magnified image demonstrating the cytotoxicity of GANPs in HepG2 cancer cells. (**A**) HepG2 cells treated with IC_50_ concentration GA/C6NPs. (**B**) HepG2 cells treated with C6NPs (negative control). Using C6 (green) and PI (red) fluorescent dyes. The scale bar is 20 µm.

The GANPs demonstrated variable patterns of cellular uptake in HepG2, MDA-MB231, HT29, and MCF7 cancer cell lines. As shown in Fig. 14, the GA/C6NPs labeled with C6 green fluorescent dye were successfully uptake by the majority of the cancer cell types with condensation in the nuclear region as shown in Fig. 15A. in section B, the empty nanoparticles labeled with C6 didn’t have any of the cells stained with PI indicating non of the cells were compromised by C6 nanoparticles and the cytotoxicity shown in section A is attributed to gallic acid as shown in Fig. 15B. Figures with diminished C6 staining as in HepG2 and MCF7 were mostly attributed to the affinity of PI to the DNA of dead cells. Once PI binds with the DNA, its fluorescence increases 20–30 times masking the fluorescence intensity of C6 due to the Förster resonance energy transfer phenomena^19^. Therefore, with more dead cells due to GA/C6NPs, it will be challenging to have the real background color of C6 nanoparticles. The cell uptake study findings were consistent with the cytotoxicity study of cancer cells using the MTT reduction assay. The GA/C6NPs showed higher cellular uptake and toxicity in HepG2 and MCF7 cells by internalizing in the nucleus and cytoplasm of cancer cells. The MDA-MB231 and HT29 cells had a lower fluorescence intensity of PI attributed to the lower toxicity of GA/C6NPs.

**Figure 16:**
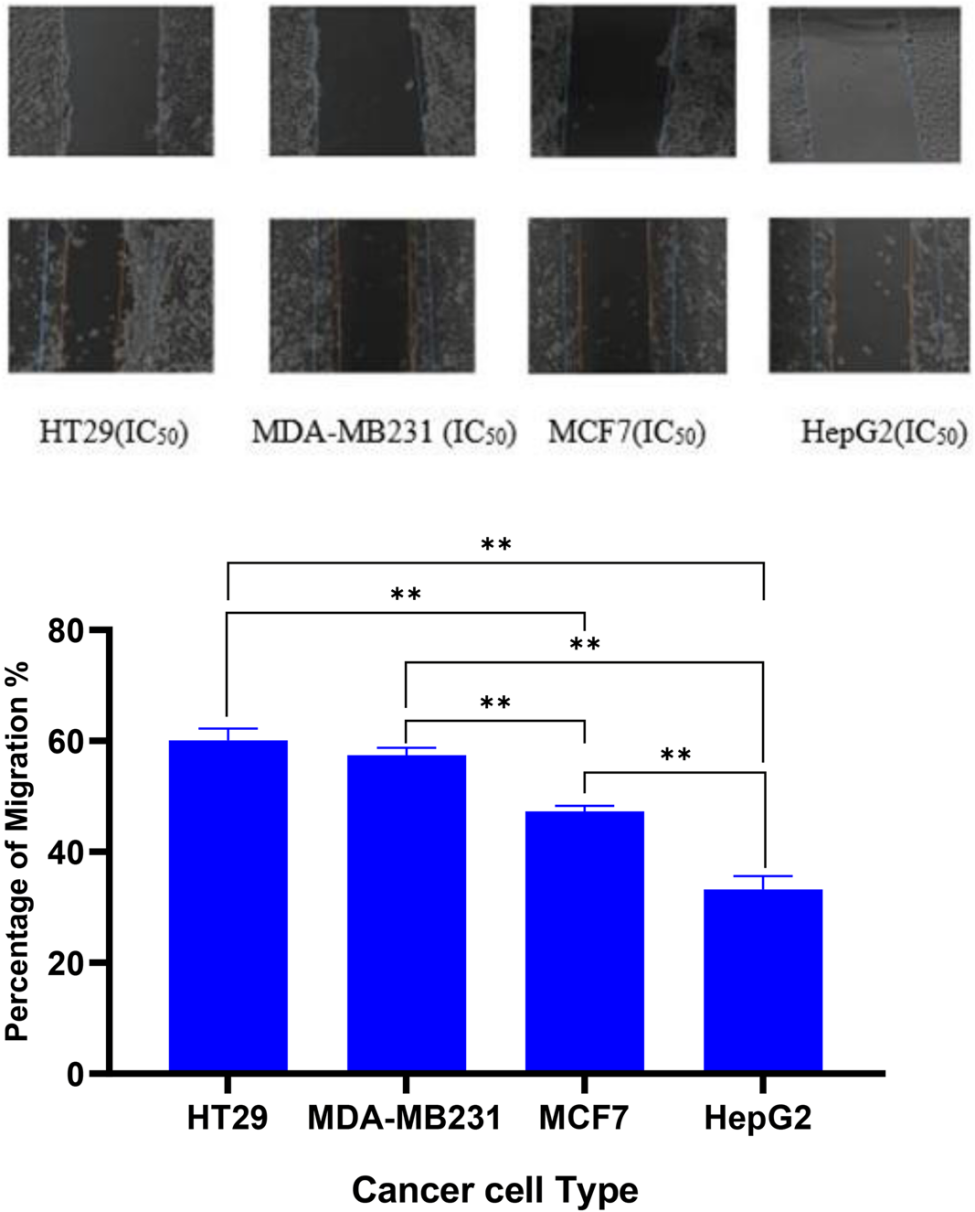
inverted microscope image (× 40 objective) magnification demonstrating the effects of GANPs on the migration of HepG2, MCF7, MDA-MB231, and HT29 cells over 24 h using scratch migration assay. The upper raw was captured immediately after creating a wound. The line indicates the initial cell-free zone with statistical analysis using a one-way ANOVA test. Data shown are mean value ± SD (n = 3, *p-value ≤ 0.05, **p-value ≤ 0.01).

The migration assay was carried out to assess the effect of GANPS and free GA with the IC_50_ value of concentrations on MCF7, MDA-MB-231, HepG2, and HT29. The pictures were captured using an inverted light microscope at 0 h and 24 h and the cell migration was calculated as percentages as illustrated in Fig. 16. The findings of this study had shown that the GANPs treatment significantly retarded the migration of HepG2 and MCF7 cells compared with MDA-MB231 and HT29 cancer cells (Fig. 16). GANPs had also demonstrated potent anti-proliferative properties in the wound zone.

## Discussion

The GANPs were prepared by the freeze-drying method with slight modifications including the high-pressure of homogenization at the pressure of 1,000 bar for 8 cycles to formulate smaller particles. Deionized water was used in the preparation to improve the dispersion of nanoparticles during the sonication process.

The X-ray diffraction technique was carried out to investigate the amalgamation between gallic acid and gum arabic as shown in Fig. 1. The physical mixture pattern as shown in the figure demonstrated GA peaks with a reduction of intensity. However, the diffraction peaks of the GANPs were completely diffused due to the amorphous state and the lack of crystallinity of the nanostructure indicating the formation of a new state in the GANPs. These changes were significantly related to the interaction between GA and gum arabic. Furthermore, the lack of a clear peak in the X-ray diffraction of GA might be the cause for the appearance of non-crystalline large peaks. Hence, the absence of crystallinity of pure compounds indicates the formation of a new phase, specifically by the conversion to the amorphous state.

In the TEM, the prepared nanoparticles had spherical elliptical shapes with the size distribution of individual particles ranging from 35 to 65 nm as shown in Fig. 4. According to the zeta sizer, the nanoparticles size measurement was in a higher range between 35 to 250 nm in aqueous solution probably due to the dry phase of TEM analysis. As demonstrated, the produced droplets had a small size to increase the surface area and the bioavailability of GA incorporated into gum arabic nanoparticles.

The use of GA is limited by its fast metabolism, low bioavailability, and poor absorption. These pharmacokinetic challenges reduce the concentration of drug reached (*Cmax*) and therefore lead to its rapid elimination^20^. Therefore, the encapsulation of GA into gum arabic nanoparticles was justified to effectively enhance its bioavailability and improve the therapeutic effects in cancer tissue by enhancing the uptake via transcellular or paracellular mechanisms^21^. Increasing the dispersion of the small nanoparticles aimed to increase the reactive surface area of gallic acid in the formulated nanoparticles^22^.

The release profile of gallic acid is depicted in Fig. 5 where the GANPs released a burst of gallic acid with a remarkably higher percentage of 41.39% at pH 4.8 compared to 25.66% at pH 7.4 after 2,000 min. This release pattern may be attributed to the weak bonds between gallic acid and gum arabic that are prone to break faster in acidic pH. This feature might offer a therapeutic privilege in cancer tissue by enhancing the release of gallic acid among cancer cells. These cells are continuously exposed to rapid shifts in the acid–base balance due to the limited blood supply exposing them more to acidic pH^23^. There was also a slow release of GA from GANPs within 3,500 min at pH 4.8 and pH 7.4. The release of GA was relatively slow at this phase compared with the initial release pattern. This pattern of release is probably due to the diminution of GA content in gum arabic nanoparticles. Hence, there was a plateau stage that lasted till 4,000 min at pH 4.8 and pH 7.4, in which about of 95.96% and 74.56% of GA was released from GANPs at pH level of 4.8 and pH 7.4, respectively. The release behavior of GA from GANPs was dependent on the negative charge of phosphate anion which gave a higher affinity for ion exchange with GA as an encapsulated agent. At pH 7.4, solubility decreased with an increase in particle binding properties due to the ionization properties of gum arabic. These findings are consistent with other studies in which the gum arabic polymer tended to disturb NPs at pH close to and higher than 5.5^22^.

Antioxidants play a crucial role in the protection against the attack of free radicals thus helping to prevent cardiovascular and cancer diseases which represent the most common causes of mortality. Oxidative stress significantly contributes to the etiology of these diseases through multiple pathways. Therefore, it is vital to keep the physiological balance between antioxidants and free radicals through the administration of potent antioxidants. Therefore, several in vitro assays were used in this study to confirm the antioxidant properties of GANPs.

The DPPH test was based on the reduction of the purple-colored stable free radical DPPH to the yellow diphenylpicrylhydrazine in the presence of free gallic acid and GANPs. Due to the existence of hydrogen-donating properties of GANPs, the phenolic compound has been shown to quench oxygen derived from free radicals by donating an electron or hydrogen atom to the free radicals in various systems from in vitro studies^24,25^. Trolox was used as a positive control to calculate the percentage of DPPH inhibition. Interestingly, GANPs nanoparticles and gallic acid exhibited an in vitro scavenging activity in a dose-dependent manner at the concentrations gradient of 50–200 µg/mL in DPPH as depicted in Fig. 6. This property of nanostructured gallic acid was reported in the literature^26^ in which the underlying mechanism of DPPH radical scavenging activity was attributed to the Single Electron Transfert (SET) and Hydrogen Atom Transfer (HAT)^26^. SET refers to a rearrangement of structure with a loss of a proton. Usually, this mechanism produces quinone derivatives, resulting in the formation of a double bond with a change of proton signals. The gallic acid is stabilized after the reaction as a radical. In contrast, HAT is attributed to the loss of proton with the stabilization action of nearby groups on the charge of the molecule. The quick proton exchange is considered as a contraction of the proton signals and it doesn’t indicate any change in the chemical structure of the molecules^27^. Although the shown antioxidant effect of GA was lower than that of Trolox, the formulated nanoparticles form had significantly enhanced these effects compared with free gallic acid due to the DPPH scavenging properties of gallic acid combined with the hydroxyl units of gum arabic. From the data in Fig. 6, it is apparent that the DPPH test has examined the impact of free radical on test compounds. Therefore, the strong absorption at 517 nm in visible spectroscopy was influenced by the odd electron of DPPH. The odd electron was paired in the presence of hydrogen of a free radical scavenger, indicating the capacity of gallic acid nanoparticles to scavenge free radicals without prior enzymatic activity^28^. The antioxidant properties of gum arabic may be attributed to its hydroxyl groups, polypeptide, and its highly branched structure which may improve the stability and antioxidant properties of gallic acid within the nanoparticles^17^. Gum arabic is, therefore, a suitable encapsulant capable of forming stable nanoparticles for safe delivery and can be used as a potent antioxidant in future formulations.

The in vitro cytotoxicity effect of the free GA and GANPs in RAW 264.7 cells was evaluated using MTT assay as demonstrated in Fig. 7. The MTT results which reflect the number of viable cells confirmed that both GANPs and GA did not have a significant cytotoxic effect on the RAW 264.7 cells. In these cells, another antioxidant test was performed known as the nitric oxide inhibition assay which is important for evaluating the antioxidant and anti-inflammatory properties of potential agents. The assay is based on the production of nitrite metabolite from Nitric oxide and its quantification using Griess reagent^29^. Nitric oxide is an important molecule in the regulation of numerous physiological mechanisms such as blood pressure and pathological conditions such as inflammation, shock and neurotoxicity^30^. Due to the scavenging capacities of antioxidants, these agents are used to treat those deleterious disorders. The cells were challenged with LPS which induces the expression of nitric oxide synthase protein. This enzyme in turn will enhance the production of nitric oxide from sodium nitroprusside and oxygen that are also induced by the oxidative challenge of LPS. The production of peroxynitrite anion, a strong oxidant is based on the interactions between superoxide radical and NO^29,31^.

The results of the nitric oxide inhibition assay confirmed significant antioxidant effects in RAW 264.7 cells. The free and nano encapsulated gallic acid at various concentrations accordingly inhibited the production of nitrite radical in the culture medium in a dose-dependent manner. As depicted in Fig. 8, the hydroxyl radical scavenging activity of GANPs was 2 times higher than that of GA at concentrations ranging between 31.5 and 500 µg/mL with IC_50_ values of 56.03 µg/mL and 105.53 µg/mL, respectively (*P* < 0.05). This result also confirmed that gallic acid maintained its antioxidant properties that were consistent with other reports^32^. Gallic acid was reported to have cyclooxygenase 2 (COX-2) and nitric oxide synthase (iNOS) inhibitory properties in LPS-induced RAW 264.7 cells modulating the pro-inflammatory cytokines such as IL-17, IFN-γ, and TNF-α^33^. The GANPs nanoparticles exhibited strong inhibition on NO production at concentrations ranging from 62.5 to 500 µg/mL. Gum arabic was also reported to enhance the free radical scavenging properties of therapeutic agents by neutralizing and absorbing free radicals^34^. Nitric oxide produced as a proinflammatory mediator during the immunopathological phenomenon may cause chronic inflammation that is circumvented by macrophages. These cells were enhanced by gum arabic to exhibit antioxidant and hepatoprotective properties^35^.

The β-carotene bleaching assay is a quick and simple test based on the competition between GANPs and β-carotene to neutralize the hydroperoxide- derived free radicals produced by the oxidation of linoleic acid. As shown in Fig. 9, a concentration-dependent inhibition of β-carotene bleaching was recorded due to the presence of phenolic groups in gallic acid. The highest reducing power was recorded at the concentration of 500 µg/mL, which exhibited approximately 68.9% of antioxidant activity (*P* < 0.05). Although both GA and GANPs showed potent antioxidant activity; however, the nano encapsulated gallic acid appeared to be significantly more effective than free gallic acid. These findings may be attributed to the phenolic groups in gallic acid^36^ and the functional groups of gum arabic used as the coating material^37^.

Oxidative stress is largely implicated in the pathogenesis of hypertension disease; where it is responsible for the activation of several mechanisms leading to vasoconstriction^38^. Therefore, the in vitro antihypertensive activity of the free and nano encapsulated gallic acid was assessed by measuring the ACE-inhibitory capacity using a UV–visible spectrophotometer at 228 nm. The inhibition of ACE function is also one of the widely used therapeutic options in the hypertension disease as this enzyme is involved in the renin-angiotensin axis of blood pressure regulation which is largely involved in the pathogenesis of hypertension^39^. The ACE that is renowned now being the target of the recent coronavirus infection is the catalyst converting angiotensin I to angiotensin II in lung and kidneys^40^. This study is the first evidence confirming the ACE inhibitory activity of GANPs in addition to its antioxidant activity providing a promising treatment for hypertension disease. As shown in Fig. 10, the ACE-inhibitory capacities of gallic acid and GANPs were time-dependent in which the ACE inhibitory activity of GANPs and Gallic acid at 90 min was 69.14% and 54.12%, respectively using 100 µL of 5 µg/mL treatment solution. The use of gum arabic polymer as nanocarrier improved the antihypertensive capacity of GANPs that explains the high percentage of ACE inhibition compared to that of free gallic acid. Such antihypertensive effects were reported in animal models of hypertension disease using free gallic acid^41^ and gum arabic^42^ treatments. Several phenolic natural products including gallic acid are known to have ACE inhibitory activity but with a poorly understood mechanism^43^. The enhanced antihypertensive property GANPs could be imputed to the high amount of gallic acid released from the nanoparticles.

The evaluation of cell viability is a common assay to determine the in vitro cytotoxicity of various biomaterials. The MTT assay is largely known as a rapid quantitative and colorimetric assay to evaluate cell viability for different purposes. The assay depends on the reduction of MTT salt by mitochondrial dehydrogenases inside the viable cells. Upon reduction, the yellow tetrazolium salt changes to the corresponding purple formazan. In this study hepatocellular, colorectal, and breast adenocarcinoma cell lines were used in addition to normal epithelial cells. Figure 11 demonstrates the cytotoxic effect of GA, GANPs in these cell lines after 72 h of exposure. It has been observed that both GA and GANPs exerted potent anti-proliferative effects in cancer cells with minimal toxicity in normal epithelial cells as shown in section A of Fig. 12. As depicted, the IC_50_ values were significantly low in all the cancer cells compared with epithelial cells. These results were consistent with previous reports confirming the antineoplastic effects of gallic acid where it significantly increased apoptosis and apoptotic markers in esophageal and melanoma cancer cells by interfering with Akt/mTOR pathway with no apparent toxicity in normal cells^44, 45^. Interestingly as shown in Fig. 11, hepatocellular cancer (HepG2) and breast cancer (MCF-7) cells appeared to be more sensitive to gallic acid nanoparticles than the MDA-MB231 breast, and HT29 colorectal cancer cells. GANPs at the concentration of 15.63 µg/mL caused a reduction to 80.16% of viable HepG2 cells n compared to untreated cells (*p* < 0.05). The nanoparticles appeared to be more potent in HepG2 cells where a lower percentage of cell viability (20.11%) was recorded at (100 µg/mL) concentration compared to 25.6% of free gallic acid. These findings could be attributed to the effect of gum arabic. In one study, gum arabic was found to have a targeting specificity towards hepatic cells by its galactose groups that attach with asialoglycoprotein receptors (ASGRP) of liver cells in which the nanocarriers stimulated receptor-mediated endocytosis of the therapeutic agents^46^. Thus, the chain in gum arabic reacted as natural targeting ligands for asialoglycoprotein receptors (ASGRP) on hepatocytes, which have improved the cytotoxicity effect of gallic acid nanoparticles. Consequently, the high cytotoxicity effect of GANPs against HepG2 cells might be attributed to the galactose units of gum arabic polymer. GANPs had also demonstrated enhanced cytotoxicity in MCF7 breast cancer cells compared to MDA-MB231 breast cancer cells. This significant potency in MCF7 cancer cells may be attributed to the modulation of estrogen receptor function as reported in several studies^47–49^. These receptors are present in MCF7 cells and are potent targets approached in the treatment of breast cancer; however, the MDA-MB231cells lack these receptors. Therefore GANPs have a better selective index toward hepatic cancer cells and MCF7 breast cancer cells probably due to the chemical structure of gum arabic polymer and the selectivity toward hepatic and breast cancer cell receptors as demonstrated in Fig. 12.

The aforementioned toxicity findings were consistent with the cellular uptake study. In this assay, coumarin-6 (C6) was selected for the observation of GANPs penetration to the cells with propidium iodide (PI) counterstain that is characterized by its penetration into nonliving cells. As shown in Fig. 14, there was a significant increase in the uptake of PI among HepG2 and MCF7 cells compared with other cancer cells that may be attributed to the higher uptake of GANPs leading to more apoptosis. The high fluorescence of PI as shown in Fig. 15 masked the green color of C6 due to the Förster resonance energy transfer phenomena. As mentioned earlier these cells were more sensitive to GANPs due to the chemical structure of gum arabic as confirmed in another report where the use of gum arabic assisted the delivery of curcumin and C6 into hepatocellular carcinoma cells^50^. The enhanced uptake of PI was consistent with several reports of the proapoptotic and cell cycle arrest properties of gallic acid in cancer cells^44,45,51,52^. In these studies, the mechanism of action was revealed in the form of enhancement of caspase, endonuclease dependant pathways, extrinsic apoptotic pathways, and disruption of p27Kip1/Skp2 Complexes that are crucial in regulating cell cycle procession in addition to modulating the level of IL-8. These findings confirm the therapeutic properties and the efficient cell internalization of gallic acid nanoparticles. Thus the use of gum arabic as a carrier permit facile passage of gallic acid through the cell membrane as well as increase its solubility.

GANPs treatment was further evaluated for its effect on the migration of HepG2, HT29, MCF7, and MDA-MB231 cancer cells as a measure of invasiveness and metastasis^53^. The treatment of cells using GANPs has revealed a significant reduction in the percentage of migration of HepG2 and MCF cells. These findings are consistent with the reported antimetastatic properties of gallic acid in gastric, cervical, melanoma, and oral cancer cells that are attributed to the interference of gallic acid with NF-kappaB activity, matrix metalloproteinases, RAS-ERK and the PI3K/AKT signaling pathways^54–57^.

## Conclusion

This study is the first to report the augmenting property of gum arabic in encapsulating gallic acid improving the therapeutic outcomes of several in vitro assays. The potent antioxidant properties of gallic acid were significantly enhanced by gum arabic coating attributed to the chemical properties of both compounds; This study also revealed several antineoplastic properties offered by the nanoformulation. The GANPs enhanced the selective uptake in hepatic cells enhanced the cytotoxicity in breast cancer (+ estrogen receptor) and confirmed its proapoptotic effects. Therefore GANPs formulation is a potential candidate for the prevention and treatment of hepatic and breast cancers. Extrapolation of the in vitro cytotoxicity effects of GANPs to in vivo cytotoxicity effects demands further investigation in light of its application as a cancer chemotherapeutic agent.

## Methodology

### Materials

Gum arabic polysaccharide was bought from ENNASR company (Sudan). Gallic acid (GA) used in this study was purchased from SINAR SCIENTIFIC company (Malaysia). Dexamethasone, TROLOX, 1,1-diphenyl-2-picrylhydrazyl (DPPH), Hippuryl-histidyl-leucine sulfonamide, Angiotensin-converting enzyme, N-(1-naphtyl) Ethylenediamine Dihydrochloride, Sodium nitrite, linoleic acid, Dulbecco’s modified Eagle’s medium (DMEM), fetal bovine serum (FBS) and streptomycin were purchased from Sigma-Aldrich company (Malaysia). Tween 80, B-carotene, quercetin, and α-tocopherol were purchased from R&M company (China). HepG2, MCF7, MDA-MB231, HT2, MCF-10A, and RAW 264.7 cells were obtained from ATCC (American Type Culture Collection). Throughout all experiments, deionized water was used.

### Preparation of gallic aid nanoparticles

The GANPs were synthesized using the freeze-drying technique with few modifications. The aqueous solution containing gallic acid (GA) and gum arabic in 1:1 molar ratio was obtained by dissolving 0.05 g of GA in 50 mL deionized water containing 0.8 g/mL of gum arabic. The mixture was kept under mild agitation at room temperature for 72 h. The final suspension was subjected to a high-pressure homogenizer at a pressure of 1,000 bar for 8 cycles and was then frozen at − 80 °C. The final product was freeze-dried for 24 h at − 55 °C. In the confocal microscope study of cell uptake and apoptosis, The GANPs labeled with C6 was prepared using a modified protocol of the same freeze-drying technique as described in some reports^58^. An aqueous solution containing gallic acid (GA) and gum arabic in 1:1 molar ratio was obtained by dissolving 0.01 g of GA and 0.1 mg of coumarin-6 in 1 mL of ethanol then mixed with 0.1 g of gum arabic dissolved in 10 mL of DMSO. The mixture was kept under mild agitation at room temperature for 72 h. The final product was then freeze-dried for 24 h at − 55 °C.

### Preparation of the physical mixture

The physical mixture of gallic acid (GA) and gum arabic was performed in a 1:1 ratio as lyophilized complex. GA and gum arabic were admixed into homogeneous powder using pestle and mortar**.**

### Characterization of GANPs nanoparticles

#### X-ray diffraction (XRD)

The GANPs, gum arabic, and GA powder samples were investigated using Shimadzu refractometer, XRD 6000 (Tokyo, Japan). Powder X-ray diffraction (XRD) patterns of GANPs, gum arabic, and GA were recorded using CuK_α_ incident beam, λ = 1.5406 Å, and voltage of 30 Kv. Analysis of samples was performed at 2θ = 20°–60° and a scan speed of 2° per minute.

#### Size and zeta potential

The Zeta potential and size of GANPs were characterized using a zeta sizer (Malvern Nano-ZS-ZS, Zeeman) with dynamic size.

### Transmission electron microscopy (TEM)

This technique was used to determine the homogeneity of GANPs nanoparticles using (TEM Model CM12 Philips; Eindhoven, The Netherlands) operated at an accelerating voltage of 200 kV and a maximum magnification of 50 k times.

### Drug release properties of GANPs

The concentrations of GA released from GANPs into the buffer solution were recorded by an ultraviolet–visible spectrophotometer at regular intervals. The release was performed at room temperature by adding about 8 mg GANPs into 25 mL phosphate buffer solution at pH 7.4 and pH 4.8. Then, 0.6 g physical mixture containing GA (0.3 g) and gum arabic (0.3) was used to determine the release as described above then compare to the behavior release of GA from GANPs.

### Determination of antioxidant and cytotoxic activity

#### DPPH scavenging activity of GANPs

The antioxidant activity of GANPs was assessed by using the DPPH method prescribed in a previously reported procedure with slight modifications^59^. In a typical process, 195 µL of DPPH solution (200 µM in methanol) was added to 100 µL of each sample solution on a 96-well microplate. DPPH served as a stable radical source, while GANPs functioned as a radical scavenger. The characteristics of DPPH assay is the ability to visualize the reaction, while the absorbance rate was recorded at 517 nm to measure the percentage of light absorption and estimate the concentrations of free radicals. Trolox was used as a positive control for the antioxidant activities with standard concentrations of 50, 75, 100, 125, 150, and 200 µg/mL. The percentage of DPPH scavenging activity was calculated using the following equation:

1 $${\text{DPPH }}\;{\text{scavenging }}\;{\text{activity}}\; \, (\% ) = \, 1 \, - ({\text{A}}_{{{\text{sample}}}} - {\text{A}}_{{{\text{sample}}\;{\text{ control}}}} ){\text{/A}}_{{{\text{blank}}}} \times 100$$

Where A sample, A blank, and A sample control are the absorbance of samples, blank, and positive control, respectively.

#### Nitric-oxide radical scavenging activity

In this experiment, the murine monocytic macrophage cell line RAW 264.7 was used to evaluate the antioxidant properties of GANPs when challenged with oxidative stress. The cells were cultured in DMEM media containing 2 mM glutamine, 10% fetal bovine serum (FBS), and 100 µg/mL streptomycin. The RAW 264.7 cells were first seeded in 96-well tissue cultures plates (1 × 10^6^ cells/100 mL) with the concentration gradient of 15.6–500 µg/mL for free gallic acid and GANPs at 37 °C with 5% CO_2_ for 24 h in a humidified incubator. The cells were then challenged with 10 µg/mL of lipopolysaccharide (LPS) for 20 h to induce oxidative stress and trigger the release of Nitric oxide (NO) with its oxidation to nitrite. The NO scavenging activity of GANPs was evaluated in RAW264.7 cells by measuring the quantity of nitrite liberated into the supernatant culture media using Griess reagent [1% (w/v) sulfonamide and 0.1% (w/v) N-(1-naphtyl) ethylenediamine dihydrochloride in 2.5% (v/v) phosphoric acid]. After cell treatment with LPS, 100 µL of cell culture was mixed with 100 µL of Griess reagent into a 96-well plate and absorbance was then recorded at 540 nm. A standard curve of sodium nitrite was prepared to determine the concentration of nitrite in samples^60^.

### β-Carotene bleaching assay

The β-carotene bleaching assay is based on the autooxidation of linoleic acid and the generated free radicals capable of π-conjugation of β-carotene to decrease of the yellow color of β-carotene. The antioxidant activities of GA and GANPs were evaluated by their competition with β-carotene compound in the linoleate model system^61^. β-Carotene (2 mg) was dissolved in 10 mL of chloroform. After evaporation of chloroform via a vacuum at 40 °C, 400 mg of Tween 80, 40 mg of linoleic acid, and 100 mL of deionized water were added to the flask with vigorous shaking for 15 min. Blank emulsion, devoid of β-carotene was prepared. Aliquots (200 µL) of β-carotene emulsion were transferred into the 96-well plate containing 50 µL of GA and GANPs at various concentrations and incubated in the dark at 50 °C. Different concentrations of α-tocopherol were used as a positive control reflecting nearly complete inhibition of β-carotene bleaching. The absorbance measurements were recorded immediately at 470 nm at 20 min intervals for 100 min. The antioxidant activity (AA) was assessed using the following equation:

2 $${\text{AA}}_{0} = [1 - ({\text{A}}_{0} - {\text{A}}_{{\text{t}}} )/({\text{A}}_{{0{\text{c}}}} - {\text{A}}_{{{\text{tc}}}} )] \times 100$$

where A_0,_ A_t_, and A_0c_, A_tc_ are the absorbances of sample at t = 0, t = 100 min and absorbance of control at t = 0, t = 100 min.

### The anti-hypertensive assay using angiotensin-converting enzyme (ACE) inhibition assay

The in vitro ACE inhibition activity of gallic acid GA in gum arabic nanoparticles was assessed in vitro based on the conversion of hippuryl-histidyl-leucine to hippuric acid in the presence of ACE enzyme^62^. One hundred microliters of gallic acid GA and GANPs were mixed with ACE (25 µL, pH 8.3) and incubated at 37 °C for 5 min. Next, 3.5 Mm of hippuryl-histidyl-leucine (10 µL) was added to the mixture and incubated for 30, 60, and 90 min. The enzymatic reaction was halted after the addition of 50 µL of 3 mol/L HCI. Hence, 1 mL of ethyl acetate was added to remove the resultant hippuric acid. The solvent was evaporated at 120 °C and re-dissolved in 3 mL of 1 N of NaCl. The concentration of hippuric acid was evaluated by measuring the absorbance at 228 nm. A blank devoid of GA and GANPs was prepared at background subtraction.

### Cell culture and cytotoxicity assay

A list of cancer cell lines selected to demonstrate the antineoplastic properties using the MTT assay which is based on the enzymatic reduction of tetrazolium salt 3-(4,5-dimethylthiazol-2-yl)-2,5 diphenyltetrazolium bromide (MTT) by active living cells. The cancer cell lines were all of an epithelial type representing a list of common cancer types including colorectal adenocarcinoma (HT29), hepatocellular cancer (HepG2), breast adenocarcinoma (MDA-MB231 and MCF7), and MCF-10A breast epithelial cell lines. All the cell lines were free of mycoplasma infection and the number of passages used in the experiments ranged between (8–10). The cells were maintained in DMEM supplemented with 1% of penicillin–streptomycin and 10% of fetal bovine serum (FBS). The cells were harvested using Trypsin–EDTA solution and examined using an inverted microscope (OLYMPUS CK40). A hemocytometer was used to determine cell density. Standard solutions of GA and GANPs were prepared and dissolved to concentrations ranging between 1.56–100 µg/mL. This assay was performed as described in our previous reports to assess cytotoxicity activity of treatments in the selected cell lines^63^. Two hundred microliters of cell suspension with a density of 1 × 10^5^ cells/mL were placed into each well of 96-well plate and later incubated at 5% CO_2_ and 37 °C for 24 h. After incubation time, the medium was replenished and the cells were treated with GA and GANPs at concentrations ranging between 1.56 and 100 µg/mL for cancer cells and 15.63–1,000 µg/mL for normal cell lines to determine the IC_50_ values. Chemotherapeutic controls were also used with different concentration gradient from 0.156 to 10 µg/mL (5-fluorouracil for HT29, tamoxifen for HepG2 cells, and doxorubicin for MCF7, MCF-10A, and MDA-MB231. The last row of the 96-well plate was left for the control experiment under similar conditions. After 72 h of post-incubation, 20 µL of 5 mg/mL of MTT solution was added to each well, in which the plate was covered with aluminum foil and incubated for 4 h. The medium was removed and replaced with 100 µL DMSO to dissolve the remaining purple formazan precipitate. The absorbance was recorded using an ELISA reader at 570 nm. This colorimetric assay focused on the reduction of MTT to purple formazan by the mitochondrial enzymes of the metabolically active cancer cells^64^. The IC_50_ was calculated from the concentration of drug that inhibits 50% of cell growth. The experiment also included the MCF-10A normal breast cell lines in the MTT assay to calculate the selective index for each of the cancer cell lines. All experiments were carried out in triplicate and the outcomes are presented in standard deviation and mean values.

### Cellular uptake of GANPs

The study also included a qualitative assessment of cell uptake in HepG2, MCF7, MDA-MB231, HT29 cancer cell lines based on the use of coumarin 6 (C6) and propidium iodide (PI) fluorescent dyes. The use of both dyes in this experiment was adapted from Win et al.^65^. C6 is widely used for tracking of nanoencapsulation emitting green light at 500 nm^66^. While PI fluorescence dye was used as a counterstain selective to nonviable cells and cells with compromised cell membranes where it binds with base pairs of double-stranded DNA giving the unique red fluorescence emission while sparing viable cells with intact cell membranes^67^.

The experiment started by culturing the cancer cells on glass coverslips with a cell suspension of 10^4^ cells/cm^2^ in complete media inside the CO_2_ incubator at 37 °C for 6 h allowing cell adherence to the coverslip. The cells were then subjected to the IC_50_ values of GA/C6NPs in serum-free medium for 24 h. consequently, the cells were incubated with PBS buffer containing PI (10 µg/mL) for 10 min at room temperature after washing with PBS buffer; then washed again with PBS buffer followed by fixation with 70% ethanol in the freezer (− 20 °C) for 10 min. The coverslips were then examined using the confocal microscope with excitation wavelength 434 nm under multichannel mode.

### Scratch migration assay

The in vitro cell migration assay was performed by scratch assay to assess cell mobility following the protocol adapted from Kovarikova et al.^68^. The HepG2, MCF7, MDA-MB231, and HT29 cells were seeded in a 6-well plate with the cell density of (2 × 10^5^). Upon reaching 80% of confluence level, the scratch was carried out using a yellow pipette tip with an average diameter of 1 mm. The cells were then washed 2 times with PBS and treated with GANPs at concentrations of 1.56–100 µg/mL and were further incubated for 24 h. Based on the Dino Eye application connected to the inverted microscope, a marker line was used to select the position of the picture captured. Various pictures were captured at 0 h and 24 h after the treatment at a magnification of 40 ×. The experiment was performed in triplicate. Hence, the percentage of migration of the aforementioned cell lines was calculated using the following formula:

$$\% \;{\text{of }}\;{\text{migration }}\;{\text{of }}\;{\text{cells }} = \frac{{({\text{distance }}\;{\text{of}}\;{\text{ scratch}}\;{\text{ at}}\; 0 \;{\text{h}} - {\text{distance}}\;{\text{ of }}\;{\text{scratch }}\;{\text{at}} \; 24 \;{\text{h}})}}{{({\text{distance}}\;{\text{ of}}\;{\text{ scratch}}\;{\text{ at }}\;0\; {\text{h}}) \times 100}}$$

### Statistical analysis

*T* tests and One-way ANOVA test followed by the Holm-Sidak multiple comparisons were performed using GraphPad Prism version 8.0.1 (San Diego, California USA) to evaluate the differences among the treatment groups. A difference at p < 0.05 was considered significant.
